# CR-39 track detector for multi-MeV ion spectroscopy

**DOI:** 10.1038/s41598-017-02331-w

**Published:** 2017-05-19

**Authors:** T. W. Jeong, P. K. Singh, C. Scullion, H. Ahmed, P. Hadjisolomou, C. Jeon, H. Yun, K. F. Kakolee, M. Borghesi, S. Ter-Avetisyan

**Affiliations:** 1 Center for Relativistic Laser Science, Institute of Basic Science (IBS), Gwangju, 61005 Republic of Korea; 2 Department of Physics and Photon Science, Gwangju Institute of Science and Technology (GIST), Gwangju, 61005 Republic of Korea; 3 School of Mathematics and Physics, The Queen’s University of Belfast, Belfast, BT7 1NN UK

## Abstract

We present the characteristics of track formation on the front and rear surfaces of CR-39 produced by laser-driven protons and carbon ions. A methodological approach, based on bulk etch length, is proposed to uniquely characterize the particle tracks in CR-39, enabling comparative description of the track characteristics in different experiments. The response of CR-39 to ions is studied based on the energy dependent growth rate of the track diameter to understand the intrinsic particle stopping process within the material. A large non-uniformity in the track diameter is observed for CR-39 with thickness matching with the stopping range of particles. Simulation and experimental results show the imprint of longitudinal range straggling for energetic protons. Moreover, by exploiting the energy dependence of the track diameter, the energy resolution (*δE*/*E*) of CR-39 for few MeV protons and Carbon ion is found to be about 3%.

## Introduction

The ongoing development of ultrashort, high intensity lasers provides a perspective for a broad range of applications in the field of intense-laser driven particle acceleration^1, 2^. Many of these applications require well-characterized ion beams with controlled parameters. Therefore, development of the instrumentation for precise diagnosis of the spectral and spatial characteristics of these beams is essential. Different types of mass-spectrometers, magnetic, electrostatic and magneto-electrostatic (e.g., Thomson spectrometer^3^), have been successfully used to measure the spectral distribution of different ion species over a broad energy range. However, the spatial characteristics of the beams are generally lost in the process. In order to measure divergence, emittance, and source characteristics of an ion beam, stacks of multiple films, such as radiochromic films^4^, image plates^5^, scintillators^6^, and track detectors^7^ are generally used. These stacks provide the energy-resolved, spatial profile of the beam, they are unable to distinguish different ion species. Additionally, an absolute calibration is necessary to pursue quantitative measurements.

In ion track detectors, the shape and the size of a track depends on species, charge (*Z*) and velocity (*v*_*i*_) of the ion as well as on the stopping power of the material. CR-39, one of the most popular track detectors, provides the absolute number of particles with 100% detection efficiency provided that the particles release enough energy as they interact with it. There have been several studies on the response of CR-39 to high-energy particles^8, 9^ where the species and the energy were identified by analysing the size and shape of the tracks. In ref. 10 the methods and applications using CR-39 detectors in several important physics research areas is described.

To reveal the damaged zones in CR-39, an etching process is needed which proceeds in several “etching time” steps in order to study the formation of the tracks due to the passage of ions. However, the etching process depends on several parameters such as etching time, temperature, concentration and inherent purity of NaOH solution, which can differ from one experiment to the other, resulting in different characteristics of the tracks even for the same “etching time”. Additionally, the fluctuations in detector properties^11, 12^ even for materials from the same manufacturer can arise due to factors such as differences in the history of detector storage, age, and differences in production series. A certain amount of fluctuation can be due to inhomogeneities in detector properties even within the same production series. The reproducibility of the detector parameters is one of the most important problem for applications.

One of the most important methods for radiation measurement and analysis using CR-39 is the linear energy transfer (LET) spectrum method. The LET spectrum method can give the differential and integral fluency, absorbed dose, dose equivalent and quality factor for a radiation field. The details of the LET spectrum method using CR-39 detectors can be found in refs 13–15 and references within. Only the dose imparted to the medium in the region close to the particle trajectory is responsible for the track formation. However, the reproducibility of the detector parameters affects largely the detector response. Therefore, to obtain the LET spectrum of the radiation field an LET calibration of CR-39 by ions is necessary.

Here a methodological approach to the determination of the energy deposition of charged particle and track formation is presented which can be considered complementary to LET calibration in respect to detector response. In this approach we propose to introduce a calibration parameter, namely the “bulk etch length”, i.e. is the etched depth in the undamaged zone of the detector plate. A study of track formation in units of “bulk etch length” allows an absolute calibration of the tracks even under different etching conditions.

In this paper we present the characteristics of track formation on the front and rear surfaces of CR-39 irradiated by laser-driven protons and carbon ions. The track diameter depends on the ions’ linear energy transfer to the CR-39. Here the track diameter variation as a function of ion energy per “bulk etch lengths” is discussed. We show that the CR-39 response to ions of different energy could be simulated from a knowledge of the dimensions of the tracks region. The proton tracks on the rear side of CR-39, when its thickness is just enough to stop the accelerated particles, show a distinct feature related to the stochastic fluctuations in the energy loss of individual protons, also called straggling range. This longitudinal range of proton straggling was experimentally measured and compared with simulation results.

## Concept

The tracks in a CR-39 plate are formed along the path of the energetic particles propagating through the material. As the particle interacts with CR-39, an electronic collision cascade process, which spreads outward from the particle trajectory, breaks the molecular structures and damages the plate^16^. The exposed CR-39 is usually etched in a 6N NaOH solution at a constant temperature (70 °C~90 °C). During the etching process, the damaged zone of CR-39 from the particle impact etches faster than in the undamaged zone of the plate. This results in the generation of the tracks, generally referred as pits. The characterisation of the pits is necessary to identify the species and the energy of particles. However, the geometrical properties (diameter and shape) of the pits do not only depend on the linear energy transfer of the particle, but also on the particular CR-39 plate and on etching conditions. Therefore, to compare data analysed under different etching conditions, a set parameter is required. Since the “bulk etched length” of the undamaged surface of CR-39 can be measured in absolute terms (in µm) and is linearly dependent on etch time, we suggest to incorporate this etched length in the analysis as a unit describing the etching process. By scaling the “etch time” (T_*etch*_) to the “bulk etch length” (L_*etch*_), an absolute scale of different etching conditions can be found. Hence, pits can be uniquely characterized and a comparative description of different experimental results can be made. Additionally, to describe the etching process, the growth rate of the track diameter (Γ) is introduced, which depends on the ratio of etched track diameter (*D*_*etch*_) and the bulk etched length (L_*etch*_) of undamaged zone. The growth rate of the track diameter is proportional to the energy released by the ions in the CR-39. After the complete etching of the damaged zone, the growth rate of the track diameter becomes equivalent to that of the undamaged part (Γ = 1).

## Results and Discussion

**Figure 1:**
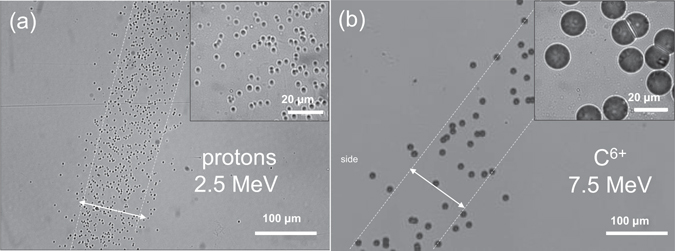
Microscopic images of the ion trace. The microscopic images (**20×** magnification) of typical particle traces of (**a**) 2.5 MeV protons and (**b**) 7.5 MeV carbon ions, on the front surface of CR-39 after 9 μm of bulk etching. The inset images are imaged with 100× magnification. The spacing between white dashed lines is equivalent to 100 μm, which is the aperture size of a pinhole.

In Fig. 1 microscope images (20× magnification) of typical particle trackes of (a) 2.5 MeV protons and (b) 7.5 MeV carbon ions on the front surface of CR-39 after 9 μm of bulk etch length are shown. The 100× magnified images displayed in the insets show a clear size difference between carbon ions (~18 μm) and protons (~2 μm).

**Figure 2:**
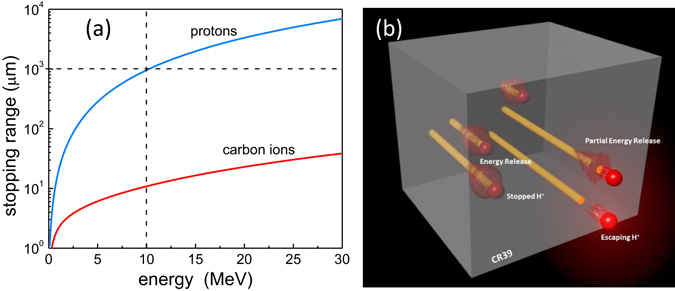
Particle stopping processes in the medium. (**a**).The stopping ranges of carbon ions and proton in CR-39 as a function of energy calculated by SRIM. The horizontal dotted line indicates that the stopping range of 10 MeV protons is equivalent to the 1 mm thickness of CR-39. (**b**) An artistic rendition depicts stopping process of protons with various energies in the CR-39 material.

The tracks formed by carbons ions were only visible on the front surface of CR-39. However, the tracks formed by protons were present on front, back, and throughout the CR-39. The stopping range of carbon ions and protons in CR-39, simulated by SRIM^17^, is shown in Fig. 2(a). The simulated stopping range of protons is orders of magnitude larger than that of carbon ions with the same energy. This is in a good agreement with the experimental findings.

Low energy protons interact with the front side of the CR-39 while high energy protons start to interact effectively with the medium only after some penetration depth. Energetic protons (~10 MeV) with stopping range of ~1 mm, do not leave any visible tracks on the front surface but only on the back surface of 1 mm thick CR-39 plate. If the ion has high enough energy, it may penetrate through a medium without releasing enough energy to damage the plate, not generating any pit. The artistic rendition of this phenomenon is shown in Fig. 2(b). The red spheres represent the protons and the yellow cylinders indicate the trajectories of the protons penetrating through CR-39. The red cloud inside the CR-39 shows the energy deposition of the protons in the CR-39 that will result in the generation of tracks. As shown in the Fig. 2(b), protons with low energies are likely to be stopped inside CR-39 while protons with high energies will penetrate through the CR-39.

### Track Characteristics for Carbon Ions

**Figure 3:**
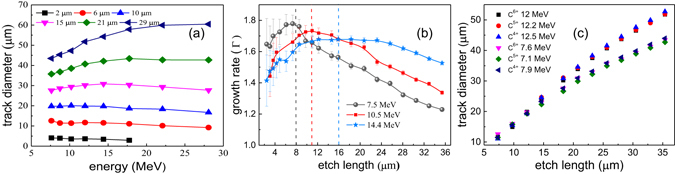
Evolution of the Carbon ion tracks. (**a**) The track diameter variation of C^6+^ for selected bulk etch length as a function of its energy. (**b**) The track diameter growth rate for 7.5 MeV, 10.2 MeV and 14.4 MeV C^6+^ energies as a function of bulk etch length. The vertical dashed lines indicate position of Bragg peak for different Carbon ion energy. (**c**) The track diameter variation of C^4+^, C^5+^ and C^6+^ for two different energies as a function of bulk etch length.

The track diameter variation of C^6+^ as a function of its energy at different bulk etch lengths (L_*etch*_) is shown in Fig. 3(a). The trends in Fig. 3(a) show that the track diameter of carbon ions remains relatively constant as a function of energy for L_*etch*_ < 15 μm while it increases with energy for L_*etch*_ > 15 μm. To understand this behavior, the growth rate of track diameter (Γ = *D*_*track*_/*L*_*track*_) for selected C^6+^ ion energies of 7.5, 10.2 and 14.4 MeV as a function of bulk etch length (L_*etch*_) is presented in Fig. 3(b). The growth rate (Γ) for all three energies is maximum for bulk etch length corresponding to their respective stopping range, shown by the vertical dotted lines. For the L_*etch*_ ≤ 8 μm, the growth rate (Γ) of low energy ion is larger than the high energy (Γ_7.5*MeV*_ < Γ_10.2*MeV*_ < Γ_14.4*MeV*_). However, for L_*etch*_ ≤ 16 μm, the trend is reversed (Γ_7.5*MeV*_ < Γ_10.2*MeV*_ < Γ_14.4*MeV*_) and the growth rate falls down with bulk etch length beyond the peak value. This is connected with the ion stopping range in the material and the etching process that reaches the damaged zone. The high growth rate of particles is linked with the bigger track diameter. For instance, while L_*etch*_ < 15 μm pits created by 7.5 MeV ion are larger than for 10.2 MeV ions. It is expected that if the etching process continues further, the growth rate of all the C^6+^ ions will asymptotically approach to the growth rate of Γ = 1, or in other words for L_*etch*_ larger than particle stopping range, the track growth rate will approach that of bulk etch rate. These imply that the track growth rate and the pit diameter depend on the ion energy, i.e. on the stopping range.

The track diameter of other charged species of carbon ions, such as C^4+^ and C^5+^, was also recorded. Bulk etch length dependence on track diameter for C^4+^, C^5+^ and C^6+^ at two different energies is shown in Fig. 3(c). Regardless of the degree of ionization, the track diameter of all the carbon species (C^4+^, C^5+^ and C^6+^) was found to be similar for a given ion energy as shown in Fig. 3(c). Therefore, the track diameter of ion is not a function of degree of ionization but is a function of energy.

### Elliptical tracks of obliquely incident Carbon ions

**Figure 4:**
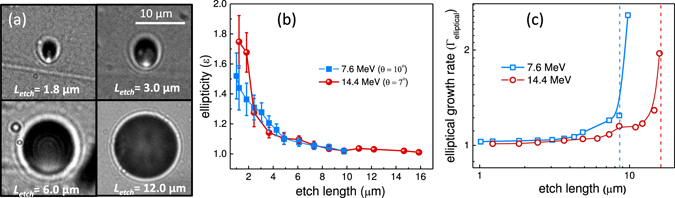
Elliptical tracks of obliquely incident Carbon ions. (**a**) Evolution of 7.6 MeV C^6+^ ion tracks changing from elliptical (for small bulk etch length) to circular (for large bulk etch length) during the etching process. (**b**) Trend of track ellipticity for 7.6 MeV and 14.4 MeV C^6+^ as a function of bulk etch length. (**c**) Bulk etch length dependent on growth rates of 7.6 MeV and 14.4 MeV C^6+^ pits derived from their angle of incidence and ellipticity. The dotted lines indicate stopping range of 7.6 MeV (blue line) and 14.4 MeV (red line) Carbon ions.

In the Thomson parabola spectrometer, the incident angle of dispersed ions on the CR-39 detector varies from about 6° to 10° for energies ranging from 22 MeV to 7.6 MeV. The shape of the track formed by the obliquely incident particle is an ellipse whose ellipticity depends on the angle of incidence, particle energy and the etch length^18^. The direction of particle incidence can be identified by the orientation of the major axis of the ellipse. For L_*etch*_ larger than the particle stopping range, the track shape gradually transforms from an ellipse to a circle. Figure 4(a) shows microscopic images of 7.6 MeV C^6+^ (incidence angle = 10°) for different bulk etched length. For small bulk etch length (L_*etch*_ < 3 µm), the tracks are highly elliptical, however for longer bulk etch length (L_*etch*_ > 6 µm), the shape becomes circular. Fig. 4(b) shows the evolution of the ellipticity (ɛ) for C^6+^ particles of two different energies, 7.6 MeV and 14.4 MeV, incident at an angle of 10° and 7° respectively.

For both energies, the ellipticity shows a continuous fall and for long bulk etching length the value approaches unity, indicating transition from elliptical to circular shape. However, it should be noted that there is not much difference in the ellipticity variation for the two energies of 7.6 MeV and 14.4 MeV. This is due to simultaneous variation of two parameters, namely, particle energy and angle of incidence. In order to resolve this, a new parameter, elliptical growth rate (Γ_*elliptical*_) has been used, which is defined as ${{\rm{\Gamma }}}_{elliptical}=\sqrt{({{\rm{\varepsilon }}}^{2}-1)/({{\rm{\varepsilon }}}^{2}\,\cos \,{\theta }^{2}-1)}$, where *θ* is incident angle of particle^19^.

The evolution of elliptical growth rate (Γ_*elliptical*_) for two energies of C^6+^ particles is plotted in Fig. 4(c). For short bulk etch length, the Γ_*elliptical*_ shows a very flat response, however as the L_*etch*_ approaches the particle stopping range (shown by the vertical dotted lines), the elliptical growth rate increases very rapidly. This behaviour is consistent with the mechanism discussed above of energy deposition by particles approaching the Bragg peak.

### Track Characteristics for Protons on front side

**Figure 5:**
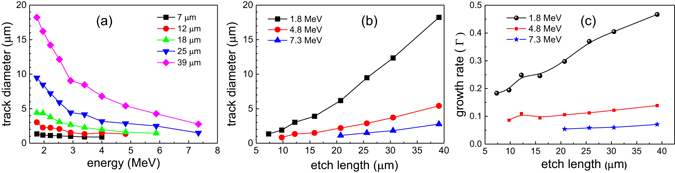
Evolution of the proton tracks. The track diameter variation of protons (**a**) for selected bulk etch length as a function of its energy and (**b**) for selected energies (1.8 MeV, 4.8 MeV and 7.3 MeV) as a function of bulk etch length. (**c**) Growth rate of the track diameter as a function of bulk etch length.

The variation of track diameter of protons as a function of energy and bulk etch length is shown in Fig. 5(a) and (b), respectively. The protons with higher energies appear at longer bulk etch length as it is shown in Fig. 5(a). For instance, 4.8 MeV protons were visible after 10 μm of bulk etch length whereas 7.3 MeV protons were visible only after 21 μm of bulk etch length. This clearly shows that in contrast to the carbon ions, the protons propagate through the medium without releasing enough energy to form a track. Since protons have stopping range nearly one order of magnitude higher than the carbon ions, their Bragg peak lies much deeper, leading to a very small energy deposition near the surface. Figure 5(b) exemplifies the track evolution for 1.8 MeV, 4.8 MeV and 7.3 MeV energy protons, and shows that new tracks of high energy particles have gradually appeared as the etching proceeds. This supports the previous discussion that the high energy particles penetrate in the material for some depth before interacting with it.

Contrary to carbon ions, the track diameter of protons decrease with energy through the entire bulk etch length (Fig. 5(a),(b)). Moreover, an increasing growth rate (Γ) of the track diameter, with respect to the bulk etch length is observed for all energies (Fig. 5(c)). The continuous increase in the growth rate (Γ) of the tracks implies that even for the lowest energy proton (1.8 MeV), the etchant has yet to reach the Bragg peak. It is expected that with further etching process (L_*etch*_ ~ 50 μm), the growth rate of 1.8 MeV proton tracks will follow similar behaviour of the carbon ion tracks.

The growth rate of carbon ions (Fig. 3(b)) is found to be much higher than that of protons (Fig. 5(c)). This is connected with the ion stopping range in the material and the etching process that has reached the damaged zone. The carbon ions are stopped on the surface of CR-39 and the bulk etch length reaches the Bragg peak while the protons are stopped inside the CR-39 and bulk etch length does not reach the Bragg peak. Other crucial parameter is deposited energy per unit length (*dE*/*dx*), which is nearly two orders of magnitude higher for carbon ions than that of protons. Strictly speaking the deposited energy per unit length is contributed to the linear energy transfer by collision of charged particles, which is the energy lost by a charged particle due to electronic collisions, minus the sum of the kinetic energies of all the electrons released with kinetic energies in excess of a threshold value (for CR-39 usually taken to be 200 eV) not contributing to the track formation^13^. Therefore, as etching process transits through the Bragg peak for carbon ions, their tracks show a much more pronounced dynamics than protons.

### Track Characteristics for Protons on rear side

From the SRIM calculation (Fig. 2), the protons with energies higher than 10 MeV have stopping range of about 1 mm in CR-39, shown by horizontal dotted line. These energetic particles penetrate through the 1 mm thick CR-39 and can only be stopped at the rear of the detector depending on their straggling range (14 μm for 10 MeV proton in CR-39). Based on the stochastic fluctuations in the energy loss of individual particle, the 10 MeV proton may escape or be stopped by 1mm thick CR-39. As discussed earlier, these protons do not deposit enough energy to generate tracks on the front side of CR-39. A previous study by Malinowska *et al*.^20^ made simultaneous observations of tracks for 6.5 MeV protons on the front and rear surface of 0.5 mm CR-39, with protons having a stopping range of 0.5 mm in CR-39. This agrees with our findings of protons with 6 MeV and 7 MeV appearing on the front side after 15 μm and 21 μm etching, respectively.

**Figure 6:**
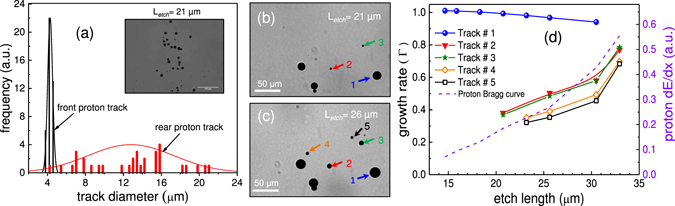
Characteristics of proton tracks appeared on the rear side of CR-39. (**a**) Distribution of the proton track diameters on the front and rear side of the CR-39. The inset shows microscopic images of 10 MeV protons appeared on the rear side of 1 mm thick CR-39. Zoomed track images of 10 MeV proton at two different bulk etching length of (**b**) 21 μm and (**c**) 26 μm. During the etching few tracks (track number 4 and 5) appear only after long bulk etch length. (**d**) The growth rate of selected proton tracks as a function of bulk etch length. The dotted line represent proton Bragg curve in CR-39.

A microscopic image of 10 MeV proton tracks, formed on the rear side of 1 mm thick CR-39, is shown in the inset of Fig. 6(a). In contrast to the low energy (E < 7.5 MeV) proton tracks observed on the front side, the rear side track diameters are not uniform even though their energies are almost the same within the resolution of the spectrometer (energy resolution of about 10 keV at 10 MeV). The much narrower distribution of front side proton tracks in comparison with rear side proton tracks is shown in Fig. 6(a). The observed differences can be understood by the probabilistic nature of the stopping process of particle, known as longitudinal range straggling. The longitudinal range straggling of protons causes particles of same energy to be stopped at different depths in CR-39. The straggling in particle stopping position can be estimated from the bulk etch length (L_*etch*_) at which tracks have appeared. The estimated longitudinal range of straggling of these protons is roughly 15 μm, which is similar to the value of 14 μm simulated by TRIM^17^.

Figure 6(b) and (c) show appearance of new tracks (Track# 4 and 5) for bulk etch length of 21 µm and 26 µm, respectively. The tracks appeared at the shortest bulk etch length, (Track#1) end up growing much larger than those who have appeared at the longest bulk etch length (Track#5). For newly revealed tracks, the bulk etch length has yet to crosse the Bragg peak, however for tracks revealed much earlier, the etching process might have crossed the Bragg peak. Therefore, the track diameter on the rear side is much larger than on the front side, where the Bragg peak has not been reached. This feature can be examined by plotting growth rate of different tracks, as shown in Fig. 6(d). As expected, for tracks which appear at short bulk etch length (Track#1), the growth rate (Γ) falls down, indicating that etching process has crossed the Bragg peak. This feature is similar to that observed for carbon ions (Fig. 3(b)) and confirms the earlier anticipation made for front side proton (Fig. 5(c)) that the growth rate for longer bulk etch length will decrease beyond the Bragg peak. For tracks that have appeared at longer bulk etch length (Track# 2–5), the growth rate increases rapidly. The violet dotted curve represents the Bragg curve for 10 MeV proton in CR-39. The qualitative similarity between Bragg curve and growth rate of Track# 2–5, indicates that etching process is approaching the Bragg peak.

**Figure 7:**
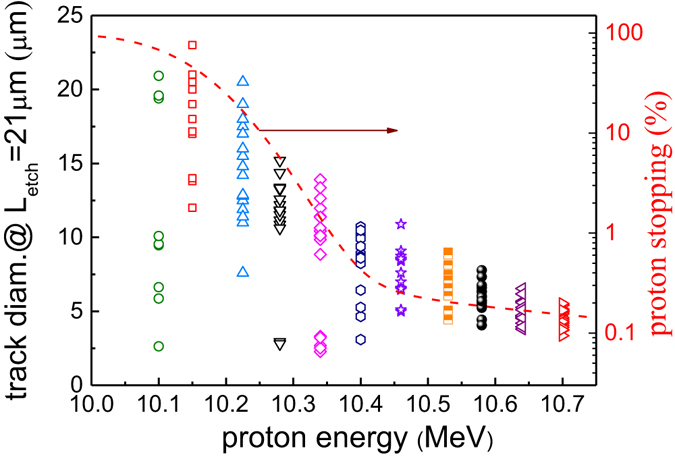
Track diameter distribution of protons in transparency regime. Track diameter and percentage of proton stopped in 1 mm thickness of CR-39, calculated by SRIM. Protons are partially transparent in the energy range of (10–10.4) MeV. The huge distribution for the track diameter implies longitudinal range straggling. The average track diameter and their distribution decrease after 10.4 MeV.

The fraction of protons stopping in 1 mm thick CR-39 calculated by TRIM and diameter of proton tracks on the rear side of CR-39 as a function of energy are shown in Fig. 7. Protons can partially pass through CR-39 at (10–10.4) MeV energy range. The large distribution of the proton track diameters in this regime implies longitudinal range of straggling. As discussed before, the protons are stopped in few µm differences within the stopping range and therefore at certain bulk etching length, some of the pits will appear in its full size and some of them will just newly appear. After this regime (*E* > 10.4 *MeV*), the mean track diameter and their distribution decreases because these protons can pass through 1 mm thick CR-39 and deposit similar amount of energy.

To further investigate the high energy protons that have penetrated through the CR-39 detector, an additional 1 mm CR-39 plate was added behind the first plate. As the protons with *E* > 10.4 *MeV* deposit enough energy to make tracks on the rear side of 1 mm thick CR-39, they are slowed down in the interim and are fully stopped in the second plate. For instance, after passing through 1 mm thick CR-39, 11 MeV protons are slowed down to 3.4 MeV. Therefore, for the protons with energies 10.4 *MeV* < *E* < 14 *MeV* in the experiments, we see same amount of tracks on the rear of the first and on the front of the second layer of CR-39. The protons with *E* > 14 *MeV* do not deposit enough energy to make tracks on the first CR-39, but still will be stopped in the second layer. The variation of track size and growth rate on the second layer of CR-39 for slowed down protons is equivalent to the same energy protons on the first layer.

Although rear tracks can be identified the longitudinal range straggling of protons causes particles of the same energy to be stopped at different depths in CR-39 resulting in non-uniform track diameters at the same particles energy. Therefore, a spectrum of particles cannot be derived only from rear tracks. Those particles can be identified using a second layer of CR-39.

### Energy resolution of the CR-39 track detectors

The energy dependent variation of track diameter in CR-39 can be used to define the energy resolution. This was demonstrated by resolving two alpha particles (6.076 and 6.119 MeV) decaying from Cf-252 source^21^. The energy resolution of the CR-39 detector is limited by the variation of the track diameter. That is for particles of two energies (*E*_*1*_ and *E*_*2*_), their respective track diameter could be *D*1 and *D*2, with uncertainty of *δD*. Therefore, the energy resolution (*δE*/*E*) can be given as

$$\frac{\delta E}{E}=\frac{({E}_{2}-{E}_{1})}{0.5\times ({E}_{2}+{E}_{1})({D}_{2}-{D}_{1})}\delta D$$

**Figure 8:**
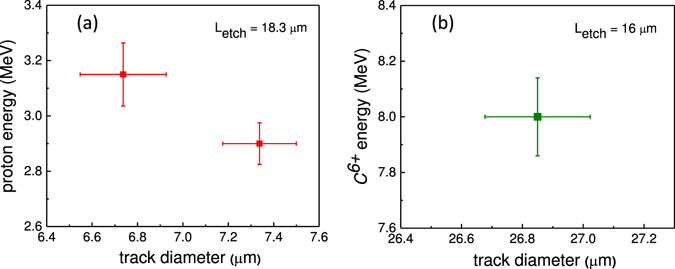
Track diameter dependent energy resolution of CR-39. Energy resolution of CR-39 for (**a**) protons nearly 3 MeV and (**b**) 8 MeV Carbon ions based on the track diameter.

By following above equation, the energy resolution (*δE*/*E*) for 2.9 MeV and 3.15 MeV is 3% and 4% respectively (Fig. 8(a)). Similarly, for Carbon ion the energy resolution (*δE*/*E*) for 8.0 MeV is nearly 2% (Fig. 8(b)). It should be noted that the CR-39 energy resolution, based on the track diameter is sensitive to bulk etch length where variation of track diameter with particle energy is more drastic (Figs 3(a) and 5(a)).

## Conclusions

In summary, the characteristics of track formation on the front and rear surfaces of 1 mm thick CR-39 produced by laser-driven protons and carbon ions are discussed based on their stopping ranges. A methodological approach is proposed to overcome the uncertainties inherently existing in etching processes. The response of CR-39 to the multispecies ion beams was studied to understand the intrinsic stopping processes of the ions within the material with respect to their Bragg peak. From the proton tracks (E > 10 MeV) on the rear surface of 1 mm thick CR-39, the longitudinal range straggling of protons was experimentally measured and compared with simulation results. By using energy dependence of the track diameter, the energy resolution (δE/E) of CR-39 for few MeV protons and Carbon ion is found to be around 3%. Once the ion spectrometry with a CR-39 is conducted, this data can be used to characterize the multispecies ion beams under same etching conditions without the aid of an additional spectrometer.

## Methods

### Experimental set-up and generation of ion beam

A Ti-Sapphire laser system^22^ at the Center for Relativistic Laser Science (CoReLS) was used to generate ion beams. A 30 fs laser pulse was focused on a 0.4 µm thick *Al* target at an incidence angle of 30° using a f/3 off-axis parabola. The laser intensity was calculated to be 2 × 10^20^ W/cm^2^ at the target surface. The trajectories of ion beams were deviated using a Thomson spectrometer with a magnetic field of ~1 T and an electric field of ~22 kV/cm. The ions were detected with the detector arrangement consist of 1 mm thick slotted CR-39 plate (from TASL, Bristol, England^9^) having identical width and spacing of 4 mm in a size of 100 × 70 mm^2^ installed in front of the MCP detector. The MCP detector was coupled with a phosphor screen, which was then imaged by a 16-bit CCD camera. This arrangement allowed the response of MCP to be calibrated with respect to the number of charged particles detected by CR-39^18^. Segmented parabolic spectral traces appeared on both MCP and CR-39 detector. Using this setup, a precise characterization of the tracks formed on the CR-39 with respect to its energy and species can be made.

### Etching procedure of the CR-39 plate

The characteristics of the tracks left on the CR-39 were revealed by etching CR-39 with 6N NaOH solution at a constant temperature of 68.5 °C. The entire etching process was carried out in small time intervals to follow evolution of the pits. After each etching step, the magnified images of the pits were recorded by a CCD connected to an optical microscope and characterised manually. The etching time step (T_*etch*_) was normalized to the bulk etch length (L_*etch*_) at the undamaged zone of the detector and it was used for further data analysis. The etching process was conducted for bulk etch length of over L_*etch*_ = 32 μm with 1 μm increments to reveal the passage of deeply stopped protons (*E* > 6 MeV).
